# Scaling Laws of Flow Rate, Vessel Blood Volume, Lengths, and Transit Times With Number of Capillaries

**DOI:** 10.3389/fphys.2018.00581

**Published:** 2018-05-23

**Authors:** Mohammad S. Razavi, Ebrahim Shirani, Ghassan S. Kassab

**Affiliations:** ^1^The George W. Woodruff School of Mechanical Engineering, Georgia Institute of Technology, Atlanta, GA, United States; ^2^The Petit Institute for Bioengineering and Bioscience, Georgia Institute of Technology, Atlanta, GA, United States; ^3^Department of Engineering, Foolad Institute of Technology, Isfahan, Iran; ^4^California Medical Innovations Institute, San Diego, CA, United States

**Keywords:** vascular design, transport, blood flow, structure-function relation, scaling laws, intraspecific scaling

## Abstract

The structure-function relation is one of the oldest hypotheses in biology and medicine; i.e., form serves function and function influences form. Here, we derive and validate form-function relations for volume, length, flow, and mean transit time in vascular trees and capillary numbers of various organs and species. We define a vessel segment as a “stem” and the vascular tree supplied by the stem as a “crown.” We demonstrate form-function relations between the number of capillaries in a vascular network and the crown volume, crown length, and blood flow that perfuses the network. The scaling laws predict an exponential relationship between crown volume and the number of capillaries with the power, λ, of 4/3 < λ < 3/2. It is also shown that blood flow rate and vessel lengths are proportional to the number of capillaries in the entire stem-crown systems. The integration of the scaling laws then results in a relation between transit time and crown length and volume. The scaling laws are both intra-specific (i.e., within vasculatures of various organs, including heart, lung, mesentery, skeletal muscle and eye) and inter-specific (i.e., across various species, including rats, cats, rabbits, pigs, hamsters, and humans). This study is fundamental to understanding the physiological structure and function of vascular trees to transport blood, with significant implications for organ health and disease.

## Significance statement

The present study reveals the simplicity of nature's proportionality laws between the form and function of a biological transport system. The mean transit time to transport blood, oxygen, nutrients, hormones, and cellular waste within the vasculature (which is fundamental to the maintenance of physiological homeostasis) is shown to scale with morphological parameters (e.g., length and diameter of blood vessels) of vascular networks. It is found that the flow needed to nourish an organ is linearly proportional to the number of capillaries needed to distribute such flow to the tissue of the organ and organism. The scaling laws hold for all organs (e.g., heart, lung, mesentery, skeletal muscle and eye) and species (e.g., rats, cats, rabbits, pigs, hamsters, humans) throughout the range of vascular dimensions (from μm to cm) for which there exist morphometric data.

## Introduction

The major role of vascular networks in the circulatory system is to transport blood, oxygen, nutrients, hormones, and cellular waste in various organs to maintain biological homeostasis. Physiological trees play a key role to transport flow to the capillary beds to support tissue demands. The tissue metabolic needs and the minimization of some specific costs for growth to maintain the delivery of nutrients and elimination of waste products generally guides the vascular development (LaBarbera, 1990).

Allometric scaling illustrates how biologic parameters vary with shape and size, regardless of the variations between organisms. Scaling laws are independent of the specific nature of an organism and originate from common underlying mechanisms. A study of the circulation requires an understanding between hemodynamic (blood flow), morphological (e.g., diameter, length, volume, etc.), and topological (e.g., connectivity patterns) information of the vasculature and any potential structure-function relations thereof. Functionally, the vascular structure serves metabolism where there is an intimate structure-function relation (LaBarbera, 1990). The vascular patterns have been used as a basis to elucidate the origin of biological allometric scaling laws (e.g., metabolic rate scaling law, West et al., 1997) and various intraspecific scaling laws (e.g., volume-diameter, flow-length, length-volume, and scaling law of flow resistance (Kassab, 2006; Huo and Kassab, 2012). For example, it is widely accepted that animal's basal metabolic rate and body mass scale to the power 3/4, known as Kleiber's law. West, Brown, and Enquist used a hemodynamic analysis of vascular networks to derive the 3/4 scaling law. Although a great majority of available empirical data comply with the 3/4 exponent, there is also statistical evidence that the 2/3 power rather 3/4 provides a better fit (Dodds et al., 2001; White and Seymour, 2003). Based on the minimum energy hypothesis, several form-form and form-function scaling relations such as volume-diameter, length-diameter, flow-length, and flow- diameter have been previously proposed and validated (Huo and Kassab, 2012). Yet, there is a need to develop scaling relations for the number of capillaries to various morphological and functional parameters based on laws of physics.

The mean transit time (MTT), which is the time required to transport blood within the vascular network, plays a vital role in the physiological function of the circulatory system (Crumrine and LaManna, 1991; Derdeyn et al., 1999). The vascular network has structural heterogeneity, the complexity of spatial arrangement of vessels and adaptation of vascular anatomy in response to hemodynamic and metabolic stimuli (Pries and Secomb, 2009). Hence, development of structure-function relations which relate the MTT to vascular morphology are fundamental to understanding the interplay between vascular form and function, and thus provide a better rationale for clinical diagnostics and therapies.

An adequate tissue perfusion (volumetric blood flow per unit mass of tissue) through a transport structure to match metabolic requirements of an organ is essential for normal function of an organism across all species. Too low of tissue perfusion may cause hypoxia, ischemia, cell death, and ultimate loss of organ function. Histological assessment of biopsy tissues, including capillary density measurements, are common but invasive and the connection with the flow and hence function is empirical and qualitative. Since there is no equivalent relation between flow and capillarity (i.e., number or density of capillaries), deriving such a relation would be of significant importance.

Here, we hypothesize the existence of scaling relations between volume, length, and flow through a branch (i.e., stem flow) of an organ's vascular system and the respective number of capillaries through which the blood distributes. Based on the scaling law of metabolic rate and fractal nature of blood vasculature, we propose and test scaling of blood volume and cumulative length of vascular networks with the respective number of terminal capillaries. We employ a one-dimensional hemodynamic analysis of an entire network incorporating the variation in blood viscosity with vessel's size (Fåhræus–Lindqvist effect) to compute blood flow. The scaling relations between capillaries, flow and cumulative length of vascular trees, in conjunction with the definition of mean transit time, provide yet another link between structure (number of capillaries) and function (mean transit time). Ultimately, we provide a form-function relation for an analytical determination of transit time based on the cumulative length and volume of vascular systems in various species and organs throughout the vasculature. The scaling laws were formulated and validated in different vascular trees (e.g., coronary, pulmonary, mesenteric vessels, skeletal muscle vasculature, and conjunctiva vessels) of various species (e.g., rats, cats, rabbits, pigs, hamsters, humans) and organs (e.g., heart, lung, mesentery, skeletal muscle, and eye) for which there exist morphometric data. The implications of the remarkably simple scaling laws are discussed in health and disease.

## Materials and methods

**Figure 1:**
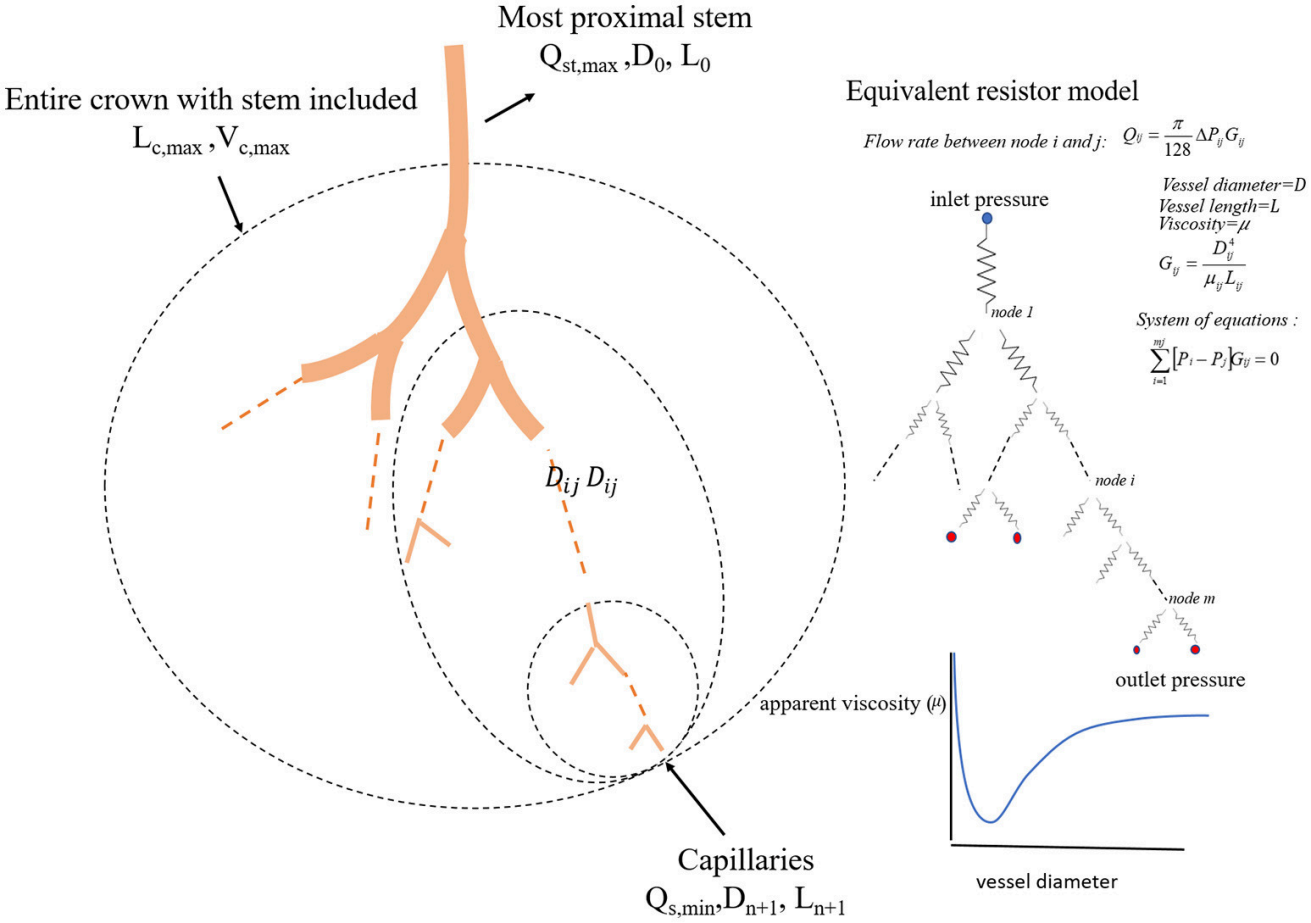
A schematic illustration of the definition of stem-crown units and the equivalent resistor model. The corresponding parameters. D, L, Q, and V are the diameter, length, flow rate, and volume, respectively. Subscriptst' “s” and “c” corresponding to stem and crown, respectively, in a stem-crown unit and subscripts “max” represent the most proximal stem-crown unit in a vascular tree.

We define a vessel segment as a “stem” and the vascular tree supplied by the stem as a “crown,” (see Figure 1). A stem-crown system in which the volume of the crown (Vc) is defined as the sum of the intravascular volume of vessel segments in the entire stem-crown system (arterial or venous trees proximal or distal to the capillaries, respectively). Similarly, the crown length (Lc) is defined as the cumulative vascular lengths in the entire arterial or venous crown. Blood flow (Q) and the number of capillaries (N) correspond to the stem and the respective network. The subscriptions *c, st*, and *cp* stand for crown, stem and capillary respectively. To derive and test the existence of various scaling laws, morphometric data based on the full asymmetric and simplified symmetric vascular system were used. The entire tree consists of many stem-crown units down to the capillary vessels (Sho et al., 2004; Huo and Kassab, 2012). At each bifurcation, there is a unique stem-crown unit which continues down to the smallest unit: an arteriole with two capillaries for an arterial tree or a venule and two capillaries for a venous tree. Functionally, each stem supplies or collects blood from the crown for an arterial or venous tree, respectively. The present analysis applies strictly to a tree structure (arterial or venous) down to the first capillary bifurcation. The entire arterial network was reconstructed down to the first capillaries (<8 μm). Missing data from the cast were reconstructed based on histological data (<40 μm) using a computational algorithm. Details of reconstruction algorithm can be found in Mittal et al. (2005). To obtain blood flow, each vessel is modeled as a resistor (Figure 1). Based on this assumption, blood vessel resistance is a function of vessel's geometry and viscosity that takes into account the Fåhræus effect. Boundary conditions were prescribed by assigning an inlet pressure of 120 mmHg and a uniform pressure of 25 mmHg at the outlet of the first capillary segment. Subsequently, a system of simultaneous linear algebraic equations for the nodal pressures is obtained. Once the vessel resistances are evaluated from the geometry, and suitable boundary conditions are prescribed, flow rate is simulated to estimate the transit time within the vascular trees (please see the Supplementary Information for details).

### Existing vascular morphometric data

Singhal S. et al. (1973); Singhal S. S. et al. (1973) and Horsfield and Gordon (1981) studied the morphometry of the pulmonary arteries and veins of humans, whereas Yen et al. (1983, 1984) used it to study the cat pulmonary arterial and venous trees. The microvasculatures of cat sartorius muscle (Koller et al., 1987), hamster retractor muscle (Ellsworth et al., 1987), hamster skin muscle (Bertuglia et al., 1991), rat mesenteric microvessels (Ley et al., 1986), rabbit omentum (Fenton and Zweifach, 1981), and human bulbar conjunctiva microvessels (Fenton and Zweifach, 1981) have also been reconstructed. Kassab et al. (Mittal et al., 2005) reconstructed the porcine right coronary artery (RCA), left anterior descending (LAD) artery, and left circumflex (LCx) arterial trees. Huang et al. (1996) measured the human pulmonary arterial and venous trees, while Jiang et al. measured the rat pulmonary arterial tree (Jiang et al., 1994).

### Data analysis

For the symmetric and asymmetric data, full tree data were presented as log-log scatter plots and log-log density plots, respectively, showing the density of data because of the enormity of data points (Huo et al., 2007). We utilized a nonlinear regression based on the least-square method and a log-log transformation to perform curve fitting of morphometric data in MATLAB, at 95% confidence level to obtain the model coefficients and confidence bounds for the fitted coefficients. R-squared and the standard error of the regression were calculated to evaluate the goodness of fit. Additionally, a nonparametric bootstrap method was used for estimating the standard error and the confidence interval of estimated parameters and correlation coefficients using repeated samples from the original data. This method was based on the sampling with replacement (Wu, 1986). A number of 1,000 bootstrap sample was used to obtain the confidence intervals of estimated parameters. Hemodynamic analyses were performed to obtain network flow based on two different models: (1) Asymmetric full model and (2) Simplified symmetric model as described in the Supplementary Information.

### Theoretical scaling laws

In this section, we propose and test different scaling relations for the crown volume (Vc), crown length (Lc), blood flow (Q), and the number of capillaries (N) in the respective network. The subscriptions *c, st*, and *cp* stand for the crown, stem and capillary respectively (please see Table 1).

**Table 1:** Variables and respective descriptions.

**Symbol**		**Description**
***V**_*c*_*	Crown volume	Cumulative volume of vessels within a stem-crown system
***L**_*c*_*	Crown length	Cumulative length of vessels within a stem-crown system
***N**_*c*_*	Crown capillary number	Number of capillaries within a stem-crown system
***T**_*c*_*	Crown transit time	Time required for blood to travel from a stem to capillaries
***Q**_*st*_*	Stem flow rate	Flow rate corresponding to a stem
***Q**_*cp*_*	Capillary flow rate	Flow rate corresponding to capillaries
***T**_*c*_*	Segment transit time	Time required for blood to travel within a vessel
***T**_*sg*_*	Segment length	Length of a vessel in stem-crown system
***L**_*cp*_*	Capillary length	The average length of capillaries
**Br**	Branching ratio	Ratio of vessel numbers in two consecutive branching levels

### Flow perfusion scales with capillary numbers

Since the structure-function relation is pervasive in biology, we hypothesize the existence of a direct relation between flow through a branch (i.e., stem flow) of an organ vascular system and the respective number of capillaries through which the blood flow distributes.

The formulation invokes the law of conservation of mass which requires the flow at the inlet of the tree or crown (*Q*_*st*_ stem flow) to be equal to the sum of the flows at the first capillary segments, *Q*_*cp*_; namely:

(1) $$Q_{st}=\sum_{i=1}^{N}Q_{cp,i}$$

where *N* is the number of capillaries perfused by a given stem. Using the average capillary flow rate $\left({\bar{Q}}_{cp}=\sum_{i}^{N}Q_{cp,i}/N\right)$, Equation (1) reduces to:

(2) $$Q_{st}=kN_{c}$$

where *k* is the average capillary flow and approximately constant across the various stem-crown systems. Hence, the inlet flow is proportional to the total number of capillary vessels. If we normalize the flow and capillarity with respect to an entire tree, we obtain the following:

(3) $$\frac{Q_{st}}{Q_{st,max}}=\left(\frac{N_{c}}{N_{c,max}}\right)$$

where *Q*_*st,max*_ and *N*_*c,max*_ are the inlet flow and the total number of capillaries in a vascular system, respectively.

#### Crown volume scales with capillary number

Crown volume is cumulative blood volume within the network $\left(Vc=\sum n_{i}(\pi L_{i}D_{i}^{2}\right)$, a derivation based on the average branching ratio (*n*_*i*_ = Br^i^, *i* = 0,., m; where n and i are number of vessels and branching level respectively) and scaling of vessel diameters and lengths in each branching level results in a relationship between crown volume and number of capillaries as follows (please see Supplementary Information):

(4) $$V_{c}=K_{VN}{\left(N_{c}\right)}^{\lambda }$$

where ***K_VN_*** is a constant. If we normalize the above equation with respect to maximum crown volume and number of capillaries in the entire vascular network, a general form of scaling relationship is obtained as:

(5) $$\frac{V_{\text{c}}}{V_{c,max}}={\left(\frac{N_{c}}{N_{c,max}}\right)}^{\lambda }$$

#### Crown length scales with capillary number

Since crown length is simply cumulative length of blood vessels of all branching levels within the network (*Lc* = ∑*n*_*i*_*L*_*i*_), a derivation based on the average branching ratio (*n*_*i*_ = Br^i^, *i* = 0,., m; where n and i are number of vessels and branching level respectively) and scaling of average length of blood vessels in each level of branching $L_{i}={\left(Br^{\gamma }\right)}^{m-i}L_{cap}$; where Lcap is the average length of capillaries and γ is an empirical exponent (Huo and Kassab, 2012), results in a direct relationship between crown length and number of capillaries (please see Supplementary Information), namely:

(6) $$L_{c}=K_{LN}N_{c}$$

where $K_{LN}-L_{cap}\overset{m}{\underset{i=o}{\sum }}Br^{\left(i-m\right)\left(1-\gamma \right)}$ is approximately a constant. A general form of normalized crown length and number of capillaries with respect to an entire tree is given as:

(7) $$\frac{L_{c}}{L_{c,max}}={\left(\frac{N_{c}}{N_{c,max}}\right)}^{\lambda }$$

where *L*_*c,max*_ and *N*_*c,max*_ are the maximum crown length and the number of capillaries in the entire tree. We shall confirm the hypothesis that λ is equal to 1 and hence the form of Equation (7) can be described by Equation (6).

#### Mean transit time scales with crown volume and length

Because of the structural heterogeneity of vascular networks and hence heterogeneous perfusions, the particles traverse various paths in the network. Hence, the mean transit time (MTT) is the average time required for blood to travel through the vascular network over a period of time. Based on the assumption that blood particles travel with the mean velocity of bulk flow and that the total number of blood particles passing through a vessel segment is proportional to the time-averaged flow rate in the segment, the MTT in the vascular network (*T*_*c*_) can be written as:

(8) $$T_{c}=\sum_{i=1}^{N}FF_{i}*T_{sg,i}$$

where *FF* is the flow fraction (ratio of segment flow to stem flow) and ^*^T_sg_ is the average transit time in a specific segment where *i* = 1,2,*., n*; and *n* is the total number of segments in the entire network. It is well known that transit time can be determined by the ratio of blood volume and blood flow (Meier and Zierler, 1954). An elementary derivation by replacing the definition of the flow fraction (FFi = Qi/Qmax), transit time in a segment (Ti = Vi/Qi) and Equation (8) results in:

(9) $$T_{c}*N_{c}=K_{TN}V_{c}$$

where *K*_*TN*_ is a proportionality constant in unit of time/volume. A combination of Equations (6) and (9) relates mean transit time to crown volume and length, namely:

(10) $$T_{c}*L_{c}=K_{TL}V_{c}$$

where the parameter *K*_*TL*_ is a proportionality constant in unit of time/area. A general normalized form of the above equation can be written as:

(11) $$\left(\frac{T_{c}}{T_{c,max}}\right)*\left(\frac{L_{c}}{L_{c,max}}\right)={\left(\frac{V_{c}}{V_{c,max}}\right)}^{\lambda }$$

where *T*_*c,max*_, *L*_*c,max*_, and *V*_*c,max*_ are the crown time, crown length and crown volume in the entire tree, respectively.

## Results

### Flow rate scales with number of capillaries

**Figure 2:**
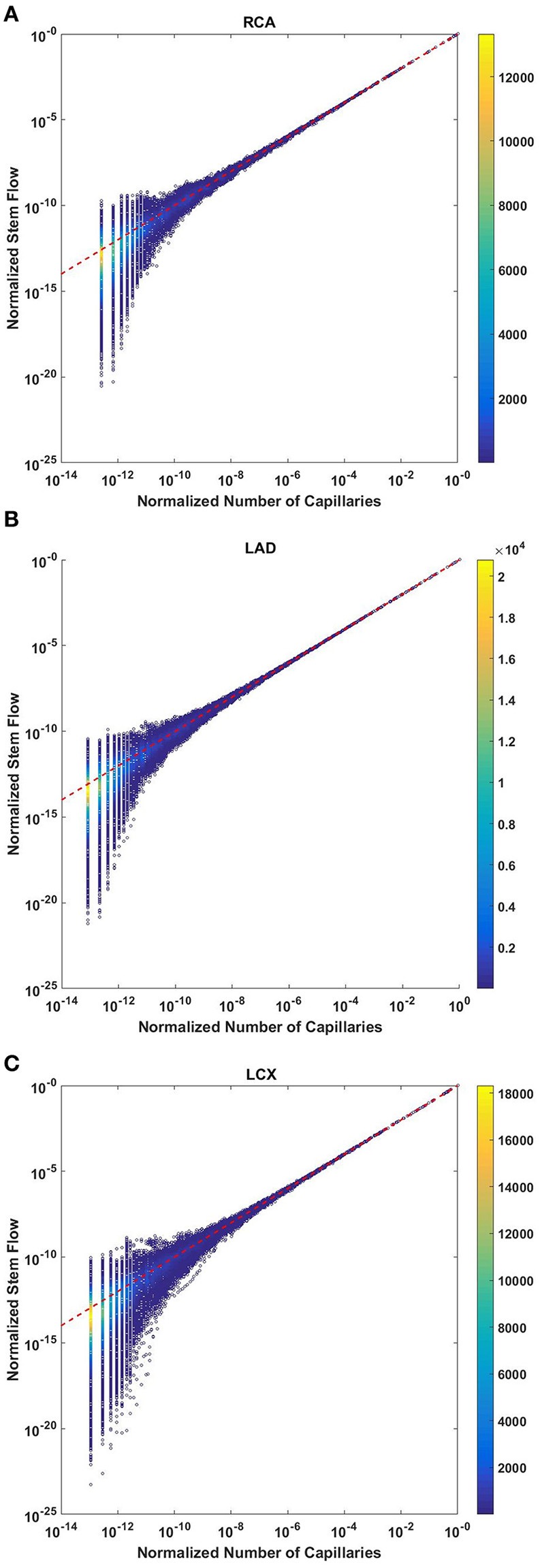
Relationship between normalized stem flow (Q_s_/Q_s,max_) and normalized number of capillaries (N_c_/N_c,max_) for the full asymmetric porcine arterial tree shown in a log-log density plot: **(A)** RCA, right coronary artery; **(B)** LAD, left anterior descending artery; **(C)** LCx, left circumflex artery. The total number of data points shown in **(A–C)** are 838,462, 950,014, and 575,868; respectively. The dash lines correspond to the theoretical exponent of unity. The values of exponents, the confidence interval and *R*^2^ for each species and organs are summarized in Table 2.

The normalized flow and number of capillaries for all stem-crown units of the full asymmetric coronary arterial trees obeys a power law (Figure 2). The values of scaling exponent λ obtained from nonlinear regression were 1.005 (*R*^2^ = 1), 1.002 (*R*^2^ = 1), and 1.005 (*R*^2^ = 1) for the porcine RCA, LAD, and LCx, respectively. The total number of data points shown in Figures 2A–C are 838,462, 950,014, and 575,868, respectively.

**Figure 3:**
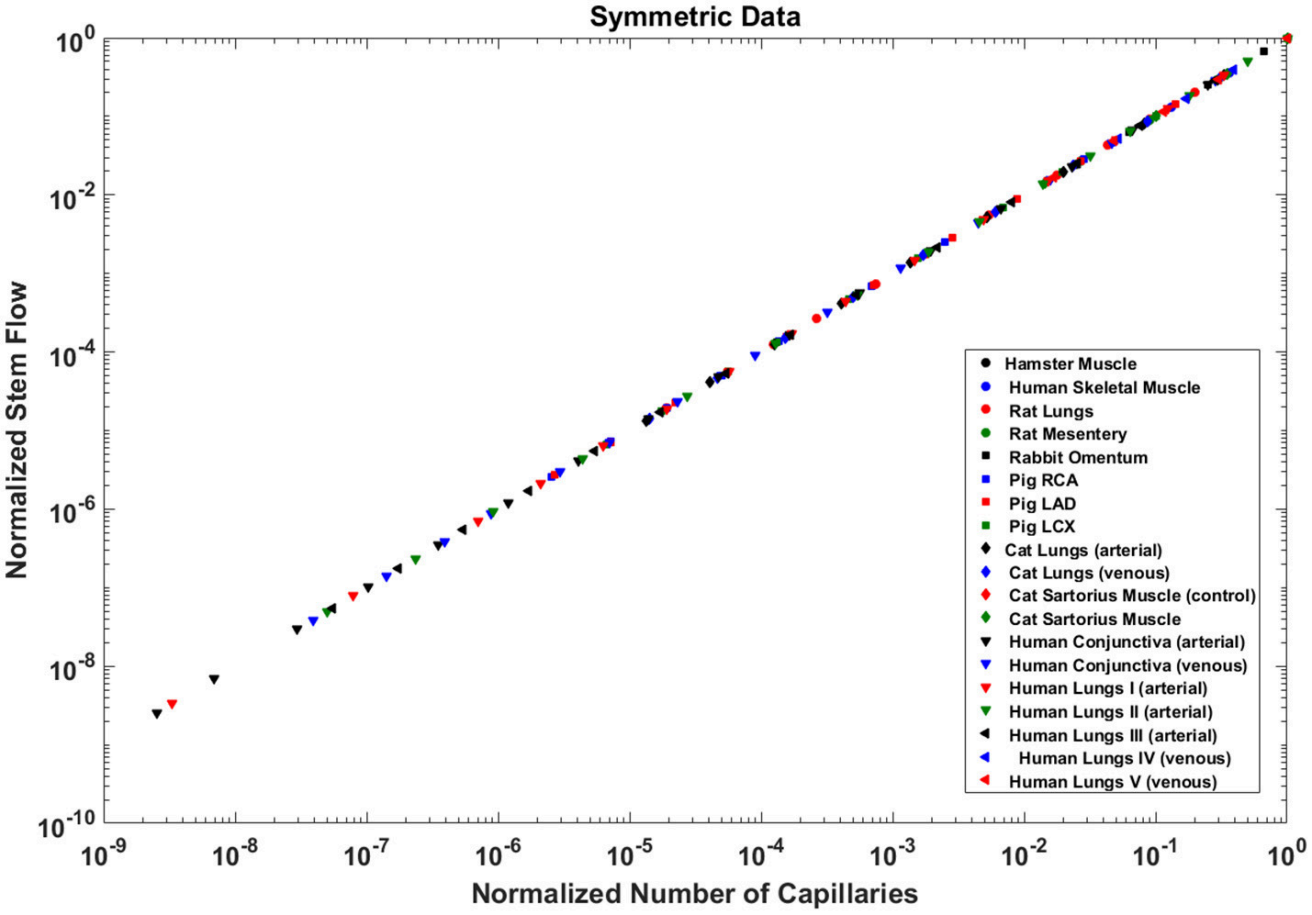
Relationship between normalized stem flow (Q_s_/Q_s,max_) and normalized number of capillaries (N_c_/N_c,max_) for the symmetric trees of various species and organs shown in a log-log scatter plot. RCA, right coronary artery; LAD, left anterior descending artery; LCx, left circumflex artery; PA, pulmonary artery; PV, pulmonary vein; SMA, sartorius muscle arteries; MA, mesentery arteries; OV, omentum veins; BCA, bulbar conjunctiva arteries; BCV, bulbar conjunctiva veins; RMA, retractor muscle artery. The values of exponents are consistent with the theoretical value of unity. The values of scaling exponents, the confidence interval and *R*^2^ for various species and organs are summarized in Table 2.

Analysis of normalized stem flow-crown capillaries for symmetric trees for various vascular trees of various species including the coronary arterial trees shows a linear relation between perfusion flow and the respective number of crown capillaries (Figure 3). The exponents in the symmetric analysis for all species and organs are equal to a theoretical value of unity; which is due to neglecting heterogeneity in the symmetric analysis and assumption that all vessel in each branching level has the same length and diameter. Table 2 summarizes the least squares power law relation for each of the vascular trees, including the coefficient, exponent, and *R*^2^. The exponents are nearly unity and the *R*^2^ is highly significant. Table 3 provides the *R*^2^values have been for lambda = 1.

**Table 2:** The validation of scaling relations in the entire stem-crown system of various – square fits.

**Species and organ**	
**Asymmetric data**	*Q* ∝ *Nc*^λ^		*Lc* ∝ *Nc*^λ^		*Vc* ∝ *Nc*^λ^		Tc^*^Lc ∝ *Vc*^λ^		**References**
	**Exponent**	***R***^2^	**Exponent**	***R***^2^	**Exponent**	***R***^2^	**Exponent**	***R***^2^	
Pig RCA	1.005 (1.004, 1.005)	0.9919	1.029 (1.029, 1.029)	0.9992	1.481 (1.481, 1.481)	0.9995	0.9814 (0.9814, 0.9814)	0.9966	Mittal et al., 2005
Pig LAD	1.002 (1.002, 1.002)	0.9937	1.031 (1.031, 1.031)	0.9993	1.453 (1.453, 1.453)	0.9995	0.9883 (0.9883, 0.9883)	0.9973	Mittal et al., 2005
Pig LCX	1.005 (1.005, 1.005)	0.9937	1.031 (1.031, 1.031)	0.9992	1.479 (1.479, 1.479)	0.9995	0.9891 (0.9891, 0.9891)	0.9972	Mittal et al., 2005
**SYMMETRIC DATA**									
Hamster Muscle	1 (1, 1)	1	1.235 (1.063, 1.407)	0.9943	1.622 (1.435, 1.808)	0.9961	0.8052 (0.693, 0.9174)	0.9093	Bertuglia et al., 1991
Rat Lungs	1 (1, 1)	1	1.033 (1.016, 1.05)	0.998	1.441 (1.418, 1.464)	0.9995	1.023 (1.012, 1.035)	0.9997	Jiang et al., 1994
Rat Mesentery	1 (1, 1)	1	1.137 (1.064, 1.211)	0.9988	1.306 (1.221, 1.391)	0.9987	1.106 (1.057, 1.156)	0.9994	Ley et al., 1986
Rabbit Omentum	1 (1, 1)	1	1.179 (1.123, 1.236)	0.9993	1.448 (1.152, 1.745)	0.9877	1.125 (1.11, 1.139)	0.9999	Fenton and Zweifach, 1981
Pig RCA	1 (1, 1)	1	1.021 (1.009, 1.032)	0.9997	1.447 (1.428, 1.467)	0.9996	1.014 (1.006, 1.022)	0.9999	Mittal et al., 2005
Pig LAD	1 (1, 1)	1	1.022 (1.009, 1.034)	0.9997	1.44 (1.408, 1.473)	0.9999	1.015 (1.007, 1.024)	0.9990	Mittal et al., 2005
Pig LCX	1 (1, 1)	1	1.028 (1.013, 1.043)	0.9996	1.506 (1.473, 1.539)	0.9991	1.019 (1.009, 1.029)	0.9998	Mittal et al., 2005
Cat Lungs (arterial)	1 (1, 1)	1	1.03 (1.02, 1.04)	0.9998	1.401 (1.339, 1.463)	0.9966	1.022 (1.016, 1.028)	0.9999	Yen et al., 1983
Cat Lungs (venous)	1 (1, 1)	1	1.029 (1.01, 1.048)	0.994	1.44 (1.414, 1.465)	0.9994	1.021 (1.008, 1.034)	0.9997	Yen et al., 1984
Cat Sartorius Muscle	1 (1, 1)	1	1.092 (1.074, 1.109)	0.9999	1.174 (1.161, 1.187)	1.0000	1.078 (1.063, 1.093)	0.9999	Koller et al., 1987
Cat Sartorius Muscle	1 (1, 1)	1	1.092 (1.074, 1.109)	0.9999	1.196 (1.176, 1.216)	0.9999	1.077 (1.061, 1.092)	0.9999	Koller et al., 1987
Human Skeletal Muscle	1 (1, 1)	1	1.43 (0.9181, 1.943)	0.9634	1.731 (1.087, 2.375)	0.9607	1.27 (1.075, 1.466)	0.9930	Ellsworth et al., 1987
Human Conjunctiva (arterial)	1 (1, 1)	1	1.105 (1.063, 1.146)	0.9993	1.227 (1.181, 1.273)	0.9993	1.086 (1.055, 1.117)	0.9996	Fenton and Zweifach, 1981
Human Conjunctiva (venous)	1 (1, 1)	1	1.113 (1.056, 1.17)	0.9986	1.406 (1.337, 1.476)	0.9987	1.081 (1.043, 1.119)	0.9994	Fenton and Zweifach, 1981
Human Lungs I (arterial)	1 (1, 1)	1	1.006 (1.001, 1.011)	0.9999	1.123 (1.114, 1.132)	0.9998	1.013 (1.009, 1.017)	0.9999	Singhal S. S. et al., 1973
Human Lungs II (arterial)	1.01 (1.003, 1.016)	0.9999	1.01 (1.003, 1.016)	0.9999	1.152 (1.124, 1.18)	0.9982	1.009 (1.004, 1.014)	0.9999	Huang et al., 1996
Human Lungs III (arterial)	1 (1, 1)	1.0000	1.002 (1.001, 1.003)	1.0000	1.197 (1.187, 1.207)	0.9998	1.005 (1.004, 1.005)	1.0000	Singhal S. et al., 1973
Human Lungs IV (venous)	1 (1, 1)	1	1.033 (1.019, 1.046)	0.9995	1.294 (1.24, 1.349)	0.9947	1.026 (1.017, 1.036)	0.9997	Huang et al., 1996
Human Lungs V (venous)	1 (1, 1)	1	1.011 (1.004, 1.019)	0.9998	1.215 (1.204, 1.225)	0.9998	1.01 (1.003, 1.016)	0.9999	Horsfield and Gordon, 1981
Mean	1.0010		1.0765		1.3723		1.0347		
*SD*	0.0024		0.1022		0.1659		0.0819		

The morphometric data were obtained from the full asymmetric arterial tree and simplified symmetric trees. The data were normalized using the maximum values in the respective tree. The least-square fit was used to obtain scaling exponent of lambda (Y = X^λ^). R^2^ is correlation coefficient; RCA, right coronary artery; LAD, left anterior descending artery; LCx, left circumflex artery; PA, pulmonary artery; PV, pulmonary vein; SMA, sartorius muscle arteries; MA, mesentery arteries; OV, omentum veins; BCA, bulbar conjunctiva arteries; BCV, bulbar conjunctiva veins; RMA, retractor muscle artery. $R^{2}=1-\frac{\sum {\left(log(y_{data})-\log(y_{fit})\right)}^{2}}{\sum {\left(\log\left(y_{data}\right)\right)}^{2}}.$

**Table 3:** The r-squared values for the hypothesized exponent λ = 1, corresponding to flow-length, length-capillary, and transit time scaling relations in stem-crown systems of symmetric data at each branching level of various species and organs, the morphometric data were obtained from the symmetric trees.

**Branching level**		**0**	**1**	**2**	**3**	**4**	**5**	**6**	**7**	**8**	**9**	**10**	**11**	**12**	**13**	**14**	**15**
Human Lungs I (arterial)	Flow-Capillary	1.000	1.000	1.000	1.000	1.000	1.000	1.000	1.000	1.000	1.000	1.000	1.000	1.000	1.000	1.000	1.000
	Length-Capillary	1.000	1.000	1.000	1.000	1.000	1.000	1.000	1.000	1.000	1.000	1.000	1.000	1.000	1.000	0.997	0.956
	Transit Time	0.988	0.999	0.998	0.997	0.998	0.999	0.999	0.999	0.999	0.999	0.999	0.998	0.997	0.995	0.986	0.873
Human Lungs II (arterial)	Flow-Capillary	1.000	1.000	1.000	1.000	1.000	1.000	1.000	1.000	1.000	1.000	1.000	1.000	1.000	1.000		
	Length-Capillary	1.000	1.000	1.000	1.000	1.000	1.000	1.000	1.000	1.000	1.000	1.000	0.998	0.995	0.987		
	Transit Time	1.000	1.000	0.998	0.999	0.998	0.998	0.998	0.996	0.986	0.974	0.979	0.937	0.946	0.931		
Human Lungs III (arterial)	Flow-Capillary	1.000	1.000	1.000	1.000	1.000	1.000	1.000	1.000	1.000	1.000	1.000	1.000	1.000	1.000	1.000	1.000
	Length-Capillary	1.000	1.000	1.000	1.000	1.000	1.000	1.000	1.000	1.000	1.000	1.000	1.000	1.000	1.000	1.000	1.000
	Transit Time	0.976	0.994	0.999	0.996	0.993	0.996	0.995	0.991	0.995	0.994	0.994	0.994	0.994	0.993	0.994	1.000
Human Lungs IV (venous)	Flow-Capillary	1.000	1.000	1.000	1.000	1.000	1.000	1.000	1.000	1.000	1.000	1.000	1.000	1.000	1.000		
	Length-Capillary	1.000	1.000	1.000	1.000	1.000	1.000	1.000	1.000	1.000	1.000	0.999	0.990	0.998	0.992		
	Transit Time	0.996	0.997	0.996	0.989	0.994	0.995	0.990	0.996	0.995	0.993	0.992	0.946	0.963	0.959		
Human Lungs V (venous)	Flow-Capillary	1.000	1.000	1.000	1.000	1.000	1.000	1.000	1.000	1.000	1.000	1.000	1.000	1.000	1.000		
	Length-Capillary	1.000	1.000	1.000	1.000	1.000	1.000	1.000	1.000	1.000	1.000	1.000	0.999	0.994	0.970		
	Transit Time	0.991	0.995	0.992	0.991	0.993	0.999	0.996	0.996	0.996	0.993	0.989	0.980	0.958	0.900		
Pig RCA	Flow-Capillary	1.000	1.000	1.000	1.000	1.000	1.000	1.000	1.000	1.000	1.000						
	Length-Capillary	1.000	1.000	1.000	1.000	1.000	1.000	0.999	0.997	0.991	0.969						
	Transit Time	0.996	0.984	0.983	0.978	0.972	0.979	0.939	0.966	0.952	0.896						
Pig LAD	Flow-Capillary	1.000	1.000	1.000	1.000	1.000	1.000	1.000	1.000	1.000	1.000						
	Length-Capillary	1.000	1.000	1.000	1.000	1.000	1.000	0.999	0.997	0.994	0.955						
	Transit Time	0.997	0.991	0.980	0.977	0.967	0.977	0.945	0.943	0.968	0.865						
Pig LCX	Flow-Capillary	1.000	1.000	1.000	1.000	1.000	1.000	1.000	1.000	1.000							
	Length-Capillary	1.000	1.000	1.000	1.000	1.000	0.999	0.996	0.994	0.955							
	Transit Time	0.997	0.984	0.966	0.978	0.957	0.943	0.938	0.969	0.865							
Cat Lungs (arterial)	Flow-Capillary	1.000	1.000	1.000	1.000	1.000	1.000	1.000	1.000	1.000							
	Length-Capillary	1.000	1.000	1.000	1.000	1.000	0.998	0.995	0.999	0.998							
	Transit Time	0.995	0.994	0.993	0.988	0.973	0.946	0.910	0.984	0.980							
Cat Lungs (venous)	Flow-Capillary	1.000	1.000	1.000	1.000	1.000	1.000	1.000	1.000	1.000							
	Length-Capillary	1.000	1.000	1.000	1.000	1.000	1.000	0.999	0.993	0.959							
	Transit Time	0.975	0.989	0.987	0.987	0.973	0.964	0.975	0.955	0.873							
Rat Lungs	Flow-Capillary	1.000	1.000	1.000	1.000	1.000	1.000	1.000	1.000	1.000	1.000						
	Length-Capillary	1.000	1.000	1.000	1.000	1.000	1.000	0.999	0.996	0.987	0.994						
	Transit Time	0.981	0.970	0.976	0.982	0.982	0.977	0.969	0.957	0.889	0.968						
Human Conjunctiva (arterial)	Flow-Capillary	1.000	1.000	1.000	1.000												
	Length-Capillary	0.999	0.997	0.994	0.981												
	Transit Time	0.991	0.979	0.972	0.933												
Human Conjunctiva (venous)	Flow-Capillary	1.000	1.000	1.000	1.000												
	Length-Capillary	0.999	0.996	0.985	0.970												
	Transit Time	0.982	0.955	0.898	0.911												
Cat Sartorius Muscle	Flow-Capillary	1.000	1.000	1.000													
	Length-Capillary	0.999	0.997	0.998													
	Transit Time	0.988	0.982	0.992													
Cat Sartorius Muscle	Flow-Capillary	1.000	1.000	1.000													
	Length-Capillary	0.999	0.997	0.998													
	Transit Time	0.990	0.984	0.992													
Human Skeletal Muscle	Flow-Capillary	1.000	1.000	1.000													
	Length-Capillary	0.997	0.974	0.815													
	Transit Time	0.977	0.954	0.770													
Rat Mesentery	Flow-Capillary	1.000	1.000	1.000													
	Length-Capillary	0.999	0.993	0.976													
	Transit Time	0.989	0.958	0.925													
Rabbit Omentum	Flow-Capillary	1.000	1.000	1.000													
	Length-Capillary	0.983	0.996	0.993													
	Transit Time	0.000	0.000	0.000													
Hamster Muscle	Flow-Capillary	1.000	1.000	1.000													
	Length-Capillary	0.998	0.988	0.935													
	Transit Time	0.970	0.940	0.869													

*The order number “0” corresponds to the main stem in the entire stem-crown systems and the last level includes terminal stem-crown system one pre-capillary branching. RCA, right coronary artery; LAD, left anterior descending artery; LCx, left circumflex artery; PA, pulmonary artery; PV, pulmonary vein; SMA, sartorius muscle arteries; MA, mesentery arteries; OV, omentum veins; BCA, bulbar conjunctiva arteries; BCV, bulbar conjunctiva veins; RMA, retractor muscle artery*.

### Crown volume scales with number of capillaries

**Figure 4:**
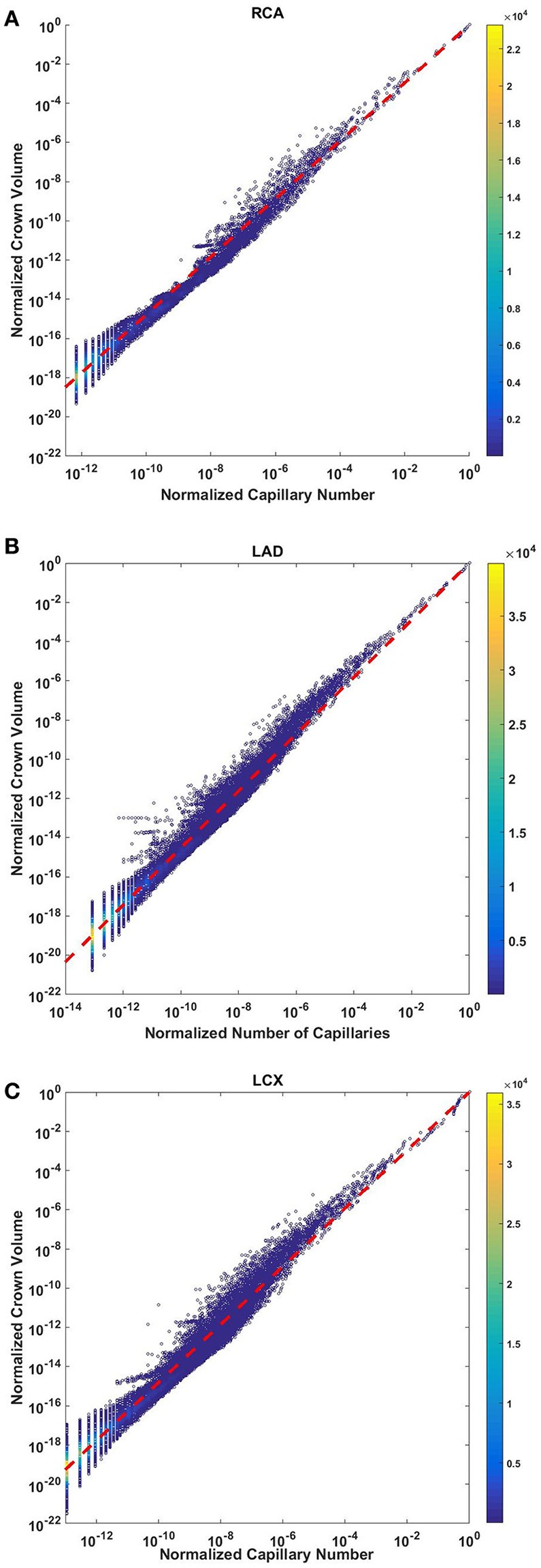
Relationship between normalized crown volume (V_c_/V_c,max_) and normalized number of capillaries (N_c_/N_c,max_) for the full asymmetric porcine arterial tree shown in a log-log density plot: **(A)** RCA, right coronary artery; **(B)** LAD, left anterior descending artery; **(C)** LCx, left circumflex artery. The total number of data points shown in **(A–C)** are 838,462, 950,014, and 575,868; respectively. The scaling exponents obtained from the least square fit of each data set are close to 3/2. The values of exponents, the confidence interval and *R*^2^ for each species and organs are summarized in Table 2.

The crown volume obeys a power law (Equation 5) as evident by morphometric data of full asymmetric arterial trees of porcine RCA, LAD, and LCx (Figure 4). The scaling exponents were 1.481 (*R*^2^ = 0.9261), 1.453 (*R*^2^ = 0.9358), and 1.479 (*R*^2^ = 0.9325). The total number of data points shown in Figures 4A–C are 838,462, 950,014, and 575,868; respectively.

**Figure 5:**
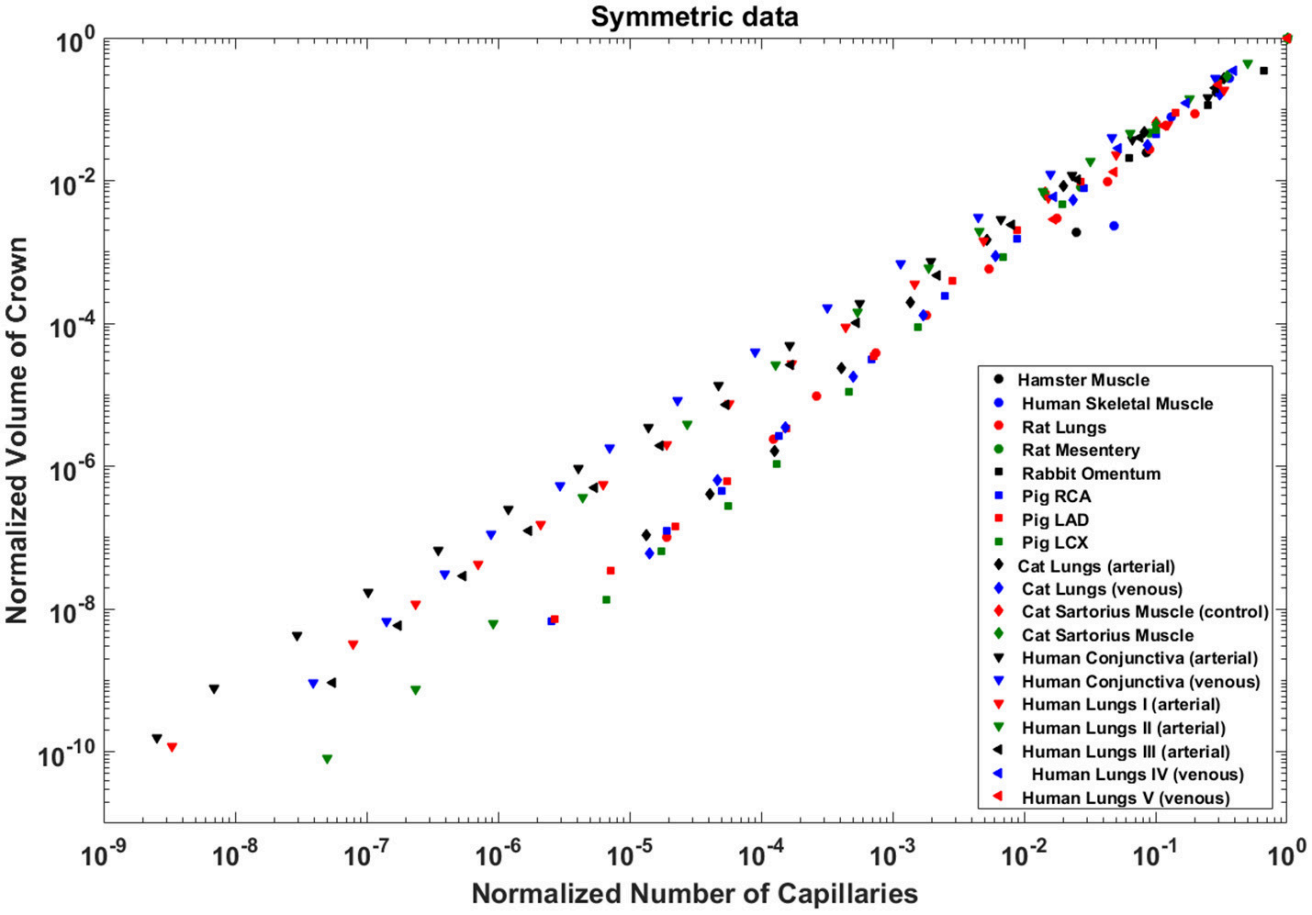
Relationship between normalized crown volume (V_c_/V_c,max_) and normalized number of capillaries (N_c_/N_c,max_) for the symmetric trees of various species and organs shown in a log-log scatter plot. RCA, right coronary artery; LAD, left anterior descending artery; LCx, left circumflex artery; PA, pulmonary artery; PV, pulmonary vein; SMA, sartorius muscle arteries; MA, mesentery arteries; OV, omentum veins; BCA, bulbular conjunctiva arteries; BCV, bulbular conjunctiva veins; RMA, retractor muscle artery. The scaling exponent (Equation 6) and *R*^2^ for each species and organs summarized in Table 2 are consistent with 3/2 or 4/3 exponent. The values of exponents, the confidence interval and *R*^2^ for each species and organs are summarized in Table 2.

The exponents in the symmetric analysis for the RCA, LAD, and LCx is 1.447 (*R*^2^ = 0.9985), 1.44 (*R*^2^ = 0.9962), and 1.506 (*R*^2^ = 0.9966), respectively; which are similar to the asymmetric tree analysis and close to the theoretical value of 3/2 (Figure 5). The mean exponent across various species and organs are 1.3723 ± 0.1659 (*R*^2^ > 0.92). The scaling exponents, confidence intervals and *R*^2^ associated with scaling exponent for various species and organs are summarized in Table 2.

### Crown length linearly scales with number of capillaries

**Figure 6:**
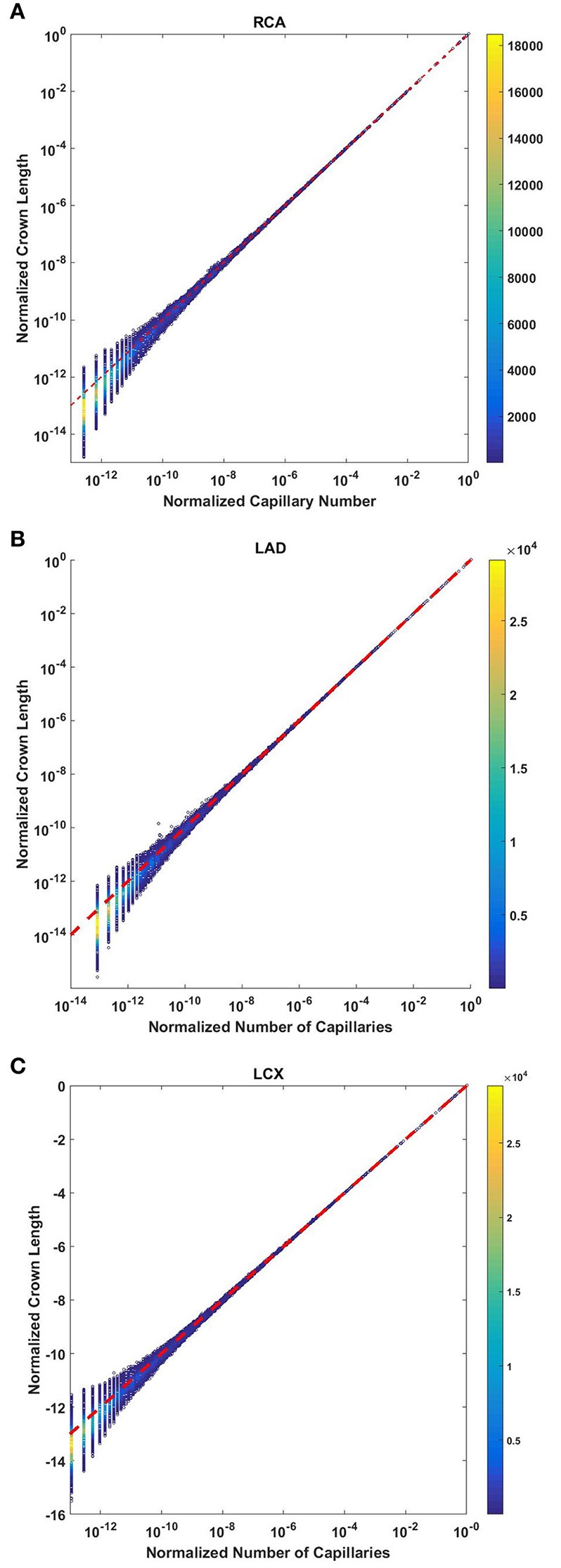
Relationship between the normalized crown length (L_c_/L_c,max_) and normalized number of capillaries (N_c_/N_c,max_) for the full asymmetric porcine arterial tree shown in a log-log scatter plot: **(A)** RCA, right coronary artery; LAD, left anterior descending artery; LCx, left circumflex artery. The total number of data points shown in **(A–C)** are 838,462, 950,014, and 575,868; respectively. The dash lines correspond to the theoretical value of 1 predicted by Equation (7). The scaling exponents obtained from the least square fit of each data set are close to the theoretical value of unity. The values of exponents, the confidence interval and *R*^2^ for each species and organ are summarized in Table 2.

The crown length linearly scales with the number of capillaries for all stem-crown units of the full asymmetric coronary arterial trees (Figure 6). The values of scaling exponent λ (Equation 7) obtained from Figures 6A–C were 1.029 (*R*^2^ = 0.9169), 1.031 (*R*^2^ = 0.9225), and 1.031 (*R*^2^ = 0.9225) for the porcine RCA, LAD and LCx, respectively (as compared to a theoretical value of unity, Equation 6). The total number of data points shown in Figures 4A–C are 838,462, 950,014, and 575,868, respectively.

**Figure 7:**
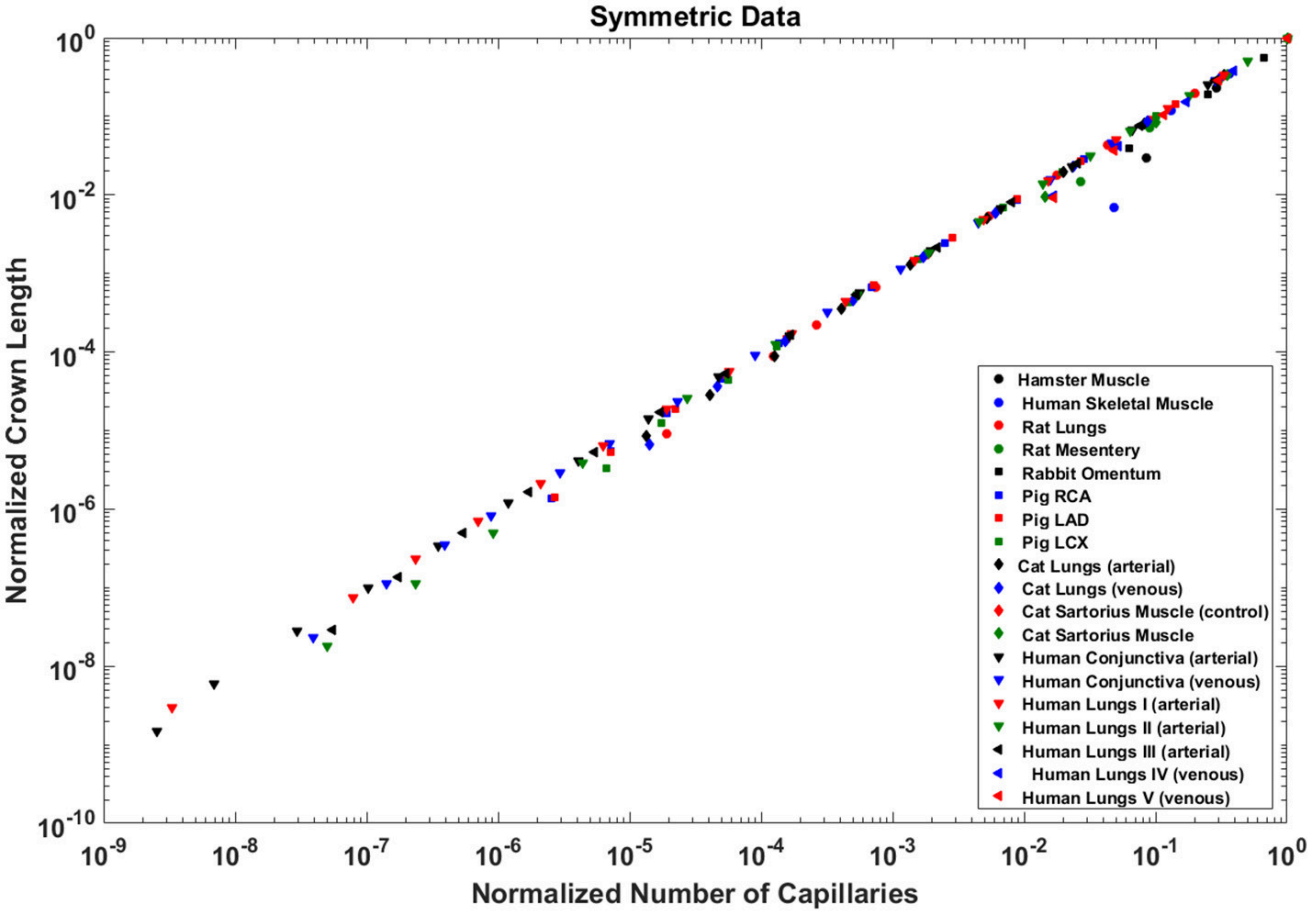
Relationship between the normalized crown length (L_c_/L_c,max_) and normalized number of capillaries (N_c_/N_c,max_) for the full asymmetric porcine arterial tree shown in a log-log scatter plot. RCA, right coronary artery; LAD, left anterior descending artery; LCx, left circumflex artery; PA, pulmonary artery; PV, pulmonary vein; SMA, sartorius muscle arteries; MA, mesentery arteries; OV, omentum veins; BCA, bulbular conjunctiva arteries; BCV, bulbular conjunctiva veins; RMA, retractor muscle artery. The values of exponents (Equation 7) are in agreement with the theoretical value of unity predicted by Equation (6). The values of scaling exponents, the confidence interval and *R*^2^ for various species and organs are summarized in Table 2.

The exponents in the symmetric analysis for the RCA, LAD, and LCx is 1.021 (*R*^2^ = 0.9989), 1.022 (*R*^2^ = 0.9988), and 1.028 (*R*^2^ = 0.9985), respectively); which are similar to the asymmetric tree analysis and close to the theoretical unity (Figure 7). The average scaling exponent (Equation 7) for all species and organs is 1.0765 ± 0.1022 (*R*^2^ > 0.9). Table 2 summarizes the least squares power law relation for each of the vascular trees, including exponent, confidence interval, and *R*^2^. The exponents are nearly unity and the *R*^2^ is highly significant.

### Transit time scales with the ratio of crown volume and length

**Figure 8:**
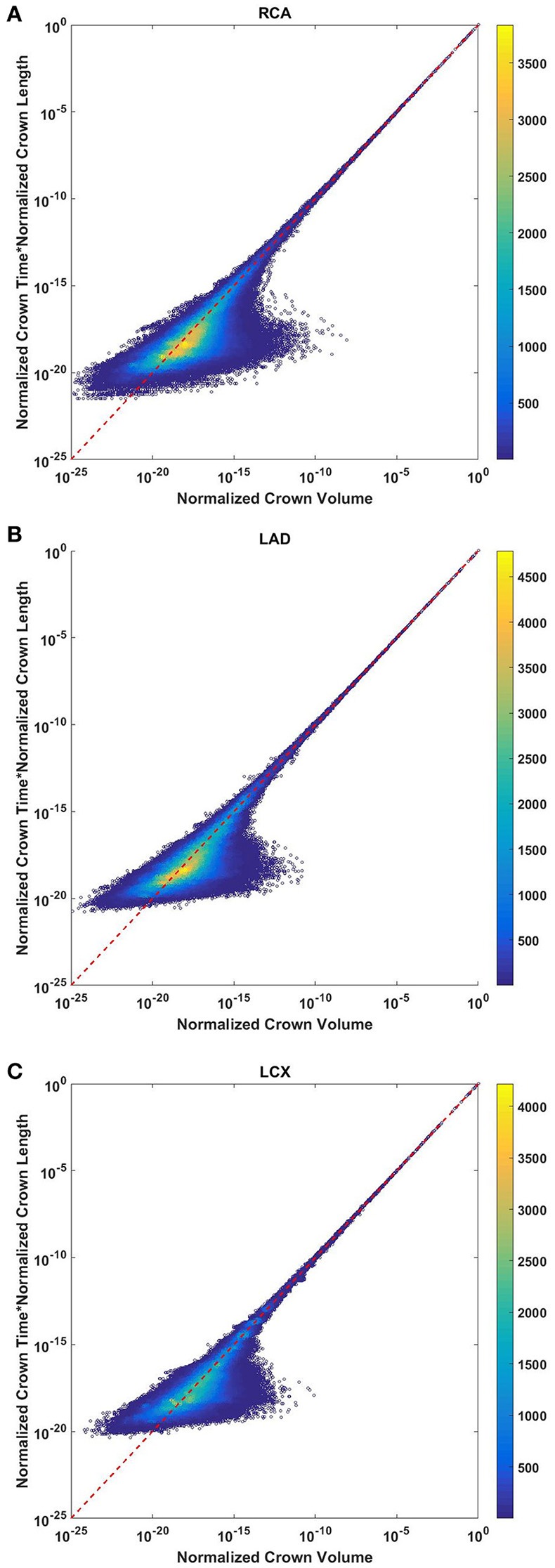
Relationship between normalized crown volume (V_c_/V_c,max_) and multiplication of crown mean transit time and crown length (T_s_/T_s,max_* L_s_/L_s,max_) for the full asymmetric porcine arterial tree shown in a log-log density plot: **(A)** RCA, right coronary artery; **(B)** LAD, left anterior descending artery; **(C)** LCx, left circumflex artery. The total number of data points shown in **(A–C)** are 838,462, 950,014, and 575,868; respectively. The dash lines correspond to the theoretical exponent of unity. The scaling exponents obtained from the least square fit of each data set are close to the theoretical value of unity. The values of exponents, the confidence interval and *R*^2^ for each species and organs are summarized in Table 2.

**Figure 9:**
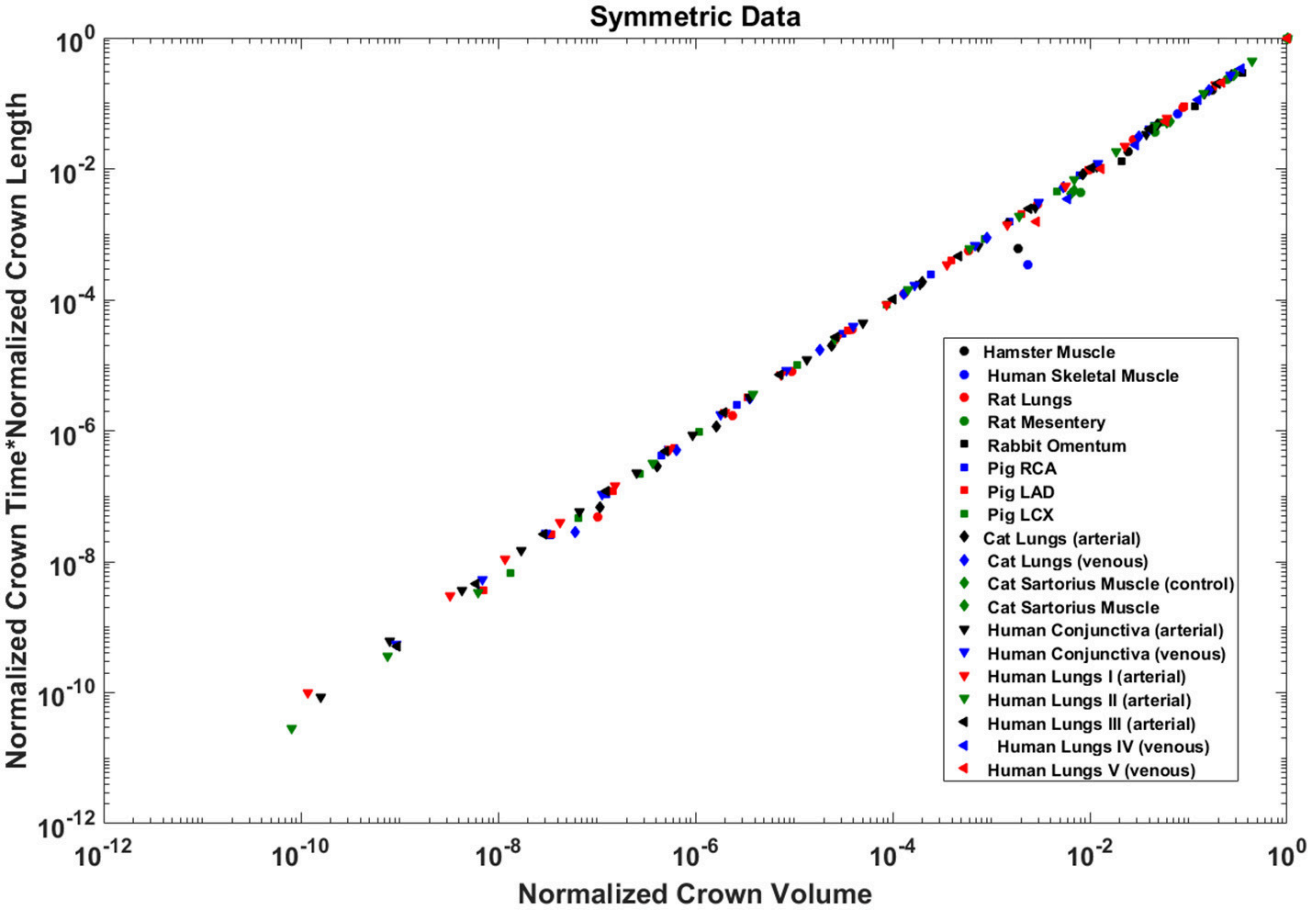
Relationship between normalized crown volume (V_c_/V_c,max_) and multiplication of crown mean transit time and crown length (T_s_/T_s,max_* L_s_/L_s,max_) for the symmetric trees of various species and organs shown in a log-log scatter plot. RCA, right coronary artery; LAD, left anterior descending artery; LCx, left circumflex artery; PA, pulmonary artery; PV, pulmonary vein; SMA, sartorius muscle arteries; MA, mesentery arteries; OV, omentum veins; BCA, bulbar conjunctiva arteries; BCV, bulbar conjunctiva veins; RMA, retractor muscle artery. The values of exponents are consistent with the theoretical value of unity. The values of scaling exponents, the confidence interval and *R*^2^ for various species and organs are summarized in Table 2.

The crown volume and the product of crown length and mean transit time of asymmetric coronary arterial trees follows a scaling relationship (Figure 8). The scaling exponent λ (Equation 11) were 0.9814 (*R*^2^ = 1), 0.9883 (*R*^2^ = 1), and 0.9891 (*R*^2^ = 1) for the porcine RCA, LAD and LCx, respectively as compared to a theoretical value of unity hypothesized by Equation (10). Similarly, the exponents in the symmetric analysis were 1.014 (*R*^2^ = 0.9995), 1.015 (*R*^2^ = 0.9995), 1.019 (*R*^2^ = 0.9994) for RCA, LAD, LCx, respectively; which are close to the exponents related to the asymmetric data (Figure 9). The mean exponents for all species and organs is 1.0347 ± 0.0819 (*R*^2^ > 0.98 for symmetric data). The exponents for various species and organs along with the associated confidence interval and *R*^2^ were summarized in Table 2.

The confidence intervals and the scaling exponents obtained from bootstrapping for the asymmetric trees confirm the results presented in this section (please see the Supplementary Information Figures S1, S2 for further details). In addition, the *R*^2^ for the stem-crown systems corresponding to a specific branching level of symmetric data where λ = 1 are presented in Table 3.

## Discussion

The scaling laws and specifically form-form and form-function relations are important theoretical tools to understand the interplay between network structure and function in physiology and pathophysiology. Among the first form-function relations, a power-law relationship between flow and diameter was first pointed out by Murray nearly 90 years ago (Murray, 1926) and came to be known as Murray's law. Murray's law has been debated and even disproven for certain organs (Hutchins et al., 1976; Uylings, 1977; Sherman, 1981; Kassab, 2006, 2007). It has been found that the exponent 7/3 provides a better fit than the theoretical power of 3 from Murray's law. Although Murray's law (i.e., the exponent of 3) has been debated, the power-law form has not been contested and is universally accepted as a consequence of the optimized design of the vascular system. To this end, key advances have been made to test and validate intra-specific and inter-specific scaling laws for the entire arterial network. The scaling laws predict a linear relationship between flow and length while volume and flow are proportional to diameter with the power of 3 and 7/3 respectively (Kassab, 2006; Huo and Kassab, 2012). It has been also shown that the form-function relations are preserved in compensatory vascular remodeling. There is no intraspecific scaling relation, however, that relates the number of capillaries to various morphological and functional parameters (Gong et al., 2016). Here, we proposed and tested scaling relations for the vascular volume, length, and flow with the number of capillaries. Although there is deviation from the theoretical lines, mainly in the small vessels, the numbers of those that deviate are relatively small compared to the very majority that concentrate near the theoretical line. It should be also noted that scatter plots for asymmetric data show the density of data points and most points are concentrated near theoretical values, and hence, the *R*^2^ values are close to one.

Here, we developed intra-specific scaling laws between capillary number and crown volume. The derivation is based on the fractal characteristic of the branching tree pattern. The scaling exponent of porcine coronary arteries is in close proximity to 3/2 while the scaling exponent for human lungs is closer to 4/3. The mean exponent across various species and organs is 1.37 ± 0.166.

The conformity between scaling of crown length and number of capillaries among various species and organs reveals another salient proportionality law between form and function of the vascular system. The blood vessels are known to adapt to physiological demands and altered homeostatic conditions. The capability of vascular trees to deliver oxygen tissue and nutrients to serve metabolism strongly depends on the number of capillaries. The capillary density changes in response to conditions like hypoxia. In the context of new blood vessel formation and vascular sprouting, length of the perfused blood vessel is a key determinant of growth and development. It has been shown that length of blood vessels adapts to changes in homeostatic conditions (Lehman et al., 1991; Sho et al., 2004; Humphrey et al., 2009). The scaling law links vascular length to respective capillaries through a structure-function relation.

A linear relationship between flow rate and capillary number is expected based on the conservation of mass. Here, we tested the internal consistency of the data for both symmetric and asymmetric trees. Although such a linear scaling law is valid if the average capillary flow across the various stem-crown system is preserved, we validated such a relationship based on the available data sets from heterogeneous vascular networks of various species and organs. Flow-length and flow-diameter relations have been previously proposed and tested based on the minimum energy hypothesis. This analysis suggests that the number of capillaries in the length-capillary relation (form-form relation) and flow-capillary relation (form-function relation) relates flow to length (Huo and Kassab, 2012). Although flow-length and flow-diameter relationships have been validated, those studies used symmetric networks to estimate flow rate. Here, we showed that steady-state simulations of blood flow through both realistic asymmetric and simplified symmetric networks confirming that flow is proportional to the number of capillaries. This analysis takes into account the effect of heterogeneity in vessel geometry and hemodynamic parameters. The physical basis of this observation is the conservation of mass that dictates the stem flow is proportional to the number of terminal capillaries as long as the average capillary flow in the various stem-crown system approximately remains similar. Further, the relative uniformity of the diameter of arterial capillaries has been previously shown by Kassab and Fung (1994) for the coronary vasculature. The coefficient of variation (CV = SD/Mean) is 0.15 and 0.18 for the right and left ventricle walls, respectively. Hence, it is well recognized that the capillary dimensions are generally conserved across species (e.g., capillary diameters are similar in rat and human (Karbowski, 2011). However, upstream blood vessels and variation of pressure at the capillary bed can lead to dispersion in the terminal flow. Hence, scaling relationships for flow-capillary, and subsequently flow-length and flow-diameter relations, provide a better fit for larger vessels where many stem-crowns are included.

Perfusion is expressed as flow per mass and hence relates proportionally to the number of capillaries per mass. Since mass is equal to the volume and density of tissue, the perfusion increases with the increase in the number of capillaries per volume of tissue or number density as can be determined histologically. Hence, the linear scaling allows a direct connection between structure (number density) and function (perfusion). This relation may be used to understand the transition between physiology and pathophysiology. When the number density of capillaries is decreased due to infarction, hypertension, or obesity, etc., this may lead to malnutrition, atrophy or death of the tissue. Conversely, the number density of capillaries may be increased in tumors in accordance with the increase in blood flow to enhance the growth of the tissue. The number density can be determined from histological sections of biopsy specimens of animals and patients.

The flow perfusion-number density scaling relation can also be used for drug dose determination. The dose can be titrated between species as the number density reflects perfusion (flow per mass) of tissue. Adequate perfusion (volumetric flow per mass of tissue) is essential for any organ because it affects its health and function. The linearity between stem flow and the number of capillaries the functional capillary density can be obtained from the length of vascular network non-invasively from standard medical imaging.

The transit time is a seminal physiological parameter in biological transport phenomena and has critical implications for vascular disease. Prolonged mean transit time is known to be associated with high risk of infarction and cerebral ischemia. No-capillary flow and altered blood volume conditions occur under pathophysiological conditions. The scaling relation between mean transit time, blood volume, and the number capillaries can be used as a theoretical basis to understand the distribution of oxygen and nutrients under physiological conditions and microvascular failure under pathological conditions. It is well known that the transit time is the ratio of vascular volume and blood flow. Since a relationship between flow rate and crown length holds for various vascular trees, we compared the theoretical calculation of transit times based on the crown length to the calculation based on the blood flow. It was shown that the estimation of transit time based on the crown length and volume hold for proximal trees (down to 1 mm diameter vessels which can be observed in angiograms). Hence, standard clinical imaging of blood vessel anatomy may yield functional data on the transit times through the organ of interest.

This study has several limitations that should be noted to guide interpretation of the proposed relationships. First, the available morphometric data were obtained from healthy subjects, and hence, the scaling laws are applicable to only healthy vasculature. For example, in pathophysiological cases such as infarction, functional capillary density and associated tissue perfusion changes even though the number of capillaries may remain unchanged. Hence, in scenarios where the model assumptions are severely violated, the proposed scaling laws may not be preserved and may lead to an overestimate. This may have utility, however, since the proposed scaling laws may serve as a signature of normal function and deviations from these laws may form the basis to quantify the severity of disease such as non-compensatory remodeling (e.g., the deviation from the scaling laws may be a useful theoretical framework to establish a scoring system for the severity of disease state). Second, a uniform outlet pressure was used to simulate blood flow. It has been found, however, that a heterogeneous outflow pressure can lead to flow reversal at capillary beds which likely occurs transiently. Although this phenomenon can change transit time estimation, previous simulations have shown that heterogeneous outlet pressure can change transit time at most ~10% (Mittal et al., 2005). We have also modeled heterogeneity of blood flow and transit time distribution incorporating Fåhræus effect in simulations. Third, both realistic asymmetric as well as idealized symmetric data were used based the available morphometric data. Since symmetric analysis neglects heterogeneity, hemodynamic variations for vessels belonging to the same branching are not considered. Specifically, that is the case for trees with a small number of branching level, where the standard deviation of each parameter in each branching level may be large. Comparison of symmetric and asymmetric analyses for trees that have a large branching level (e.g., pig RCA, LCX, LAD), however, shows that the scaling exponents are very similar for both symmetric and asymmetric data.

## Authors contribution

MR performed the analysis and drafted the text; ES reviewed the transit time analysis and provided input; GK performed the analysis for the flow-capillary number relation, drafted the related text, and supervised the overall manuscript.

### Conflict of interest statement

The authors declare that the research was conducted in the absence of any commercial or financial relationships that could be construed as a potential conflict of interest.
