# Supplementary material for: Scaling Laws of Flow Rate, Vessel Blood Volume, Lengths, and Transit Times With Number of Capillaries
Source: Front Physiol. 2018 May 23;9:581. doi: 10.3389/fphys.2018.00581 (PMC5974547; doi:10.3389/fphys.2018.00581)
Supplement: Supplementary file 1 [file Data_Sheet_1.pdf]

SUPPLEMENTARY INFORMATION

Derivation of Relationship between Crown Length and Capillary Number

The crown length is the cumulative length of blood vessels of all branching levels within the network, namely:

$$Lc = \sum n_i L_i \tag{1}$$

Based on the average branching ratio ($n_{i+1}/n_i = Br$, i=0,…,m; where n and i are number of vessels and branching level respectively) and the average length ratio (($L_{i+1}/L_i = \mathrm{Br}^{\gamma}$, where L is the average length of vessel in each branching level and γ is an empirical parameter, the average length in each branching level is written as:

$$\mathrm{L_i} = (\mathrm{Br}^{\gamma})^{m-i}\mathrm{L_{cp}} \tag{2}$$

where m and L_{cp} are the maximum branching level and the length of vessels corresponding to capillaries. A combination of Eq. (1) and Eq. (2) results in:

$$L_c = (\sum_{i=o}^{m} Br^{(i-m)(1-\gamma)})L_{cap}N_c \tag{3}$$

where N_c is the number of capillaries in the stem-crown system. Since the geometric series converges to a constant value, the above equation can be written as:

$$L_c = K_{LN}N_c \tag{4}$$

Similarly, crown volume is the cumulative volume of blood vessels of all branching levels within the network, namely:

$$V_c = \sum n_i(\frac{\pi}{4} * L_i D_i^2) \tag{5}$$

Based on the assumption that vessel length and diameter scales at each branching level ($L_{i+1}/L_i = Br^{\gamma}$ and $D_{i+1}/D_i = Br^{\alpha}$), and using branching ratio and number of capillaries ($N_c = Br^m$, where m is maximum number of branching levels), a relationship between crown volume and number of capillary is derived as follows:

$$V_c = \frac{\pi}{4} * V_{cap} \sum_{i=o}^{m} N_c{}^{(\mathrm{i}+(\mathrm{m}-\mathrm{i})(2\alpha+\gamma))/m}$$

Hence, we hypothesize a power law relationship between crown volume and capillary number in as follows:

$$\boldsymbol{V_c} \ = \boldsymbol{K_{VN}}(\boldsymbol{N_c})^{\boldsymbol{\lambda}} \tag{6}$$

where K_{LN} is an approximately constant value.

Existing Database on Morphometry of Vascular Trees. Based on available morphometric data of vascular trees in various organs and species, the transit time scaling laws are quantitatively assessed. The analysis considers a formulation for tree structures of various species and organs from the most distal vessels to the only first segment of capillary vessels, but excluding the capillary network which is not a tree-like structure. The following morphometric data bases were used to validate the scaling law formulations for transit time: coronary arterial trees of pig hearts by Kassab et al. (46), pulmonary arterial tree of rats from the study of Jiang et al. (24), pulmonary arterial/venous trees of cats from Yen et al. (15, 16), pulmonary arterial trees of humans from Singhal et al. (12, 13) and Huang et al. (23), pulmonary venous trees of humans from Horsfield and Gordon (14) and Huang et al. (23), retractor muscle arterial tree of hamsters from Ellsworth et al. (18), mesentery arterial tree of rats from Ley et al. (20), sartorius muscle arterial tree of cats from Koller et al. (17), and bulbular conjunctiva arterial/venous trees of humans and the omentum arterial tree of rabbits from Fenton and Zweifach (21). The entire arterial network was reconstructed down to the first capillaries (<8µm). Missing data from the cast were reconstructed based on histological data (<40µm) using a computational algorithm. Details of reconstruction algorithm can be found in ref. (11).

Network Hemodynamic Analysis. To validate scaling laws, we carried out a network flow analysis based on two different models: 1) Asymmetric full model and 2) Simplified symmetric model. For the first model, the asymmetric coronary arterial tree has been reconstructed in pig hearts by using the growth algorithm introduced by Mittal et al. (22) based on measured morphometric data of Kassab et al. (46). Briefly, under laminar and steady flow, the Poiseuille's law for a fluid can be stated as:

$$Q_{ij} = \frac{\pi}{128} \Delta P_{ij} G_{ij} \tag{7}$$

where Q_{ij} is the volumetric flow, in a vessel between any two nodes, represented by i and j. ΔP_{ij} is the pressure differential given by $\Delta P_{ij} = P_i - P_j$, and vessel conductance, G_{ij}, is given by $G_{ij} = \frac{D_{ij}^4}{\mu_{ij} L_{ij}}$ where D_{ij}, L_{ij}, and μ_{ij} are the diameter, length, and viscosity, respectively, between nodes i and j. The variation of apparent viscosity with vessel diameter is given by Pries et al. (47). Two or more vessels emanate from the jth node anywhere in the tree with the number of vessels converging at the jth node being m_j. If we combine conservation of mass with above equation, we obtain a set of linear algebraic equations in pressure for M nodes in the network, namely:

$$\sum_{i=1}^{mj} \left[P_i - P_j\right] G_{ij} = 0 \tag{8}$$

The system of equations reduce to a set of simultaneous linear algebraic terms for the nodal pressures once the conductances are evaluated from the geometry, and suitable boundary conditions are specified. This system is based on the assumption that each blood vessel can be modeled as a resistor while the resistance is a function of vessel's geometry. The apparent viscosity is also a function of the vessel diameter that models the Fåhræus–Lindqvist effect. Boundary conditions were prescribed by assigning an inlet pressure of 120 mmHg and a uniform pressure of 25 mmHg at the outlet of the first capillary segment. This system of equations was then solved using a General Mean Residual algorithm (48) to determine the pressure values at all internal nodes of the arterial tree. The pressure drops as well as the corresponding flows were subsequently calculated.

For the second model, we adapted a simple symmetric model that simulates the average statistical data of the trees. Physically, the symmetric model is equivalent to assuming that all the vessel elements in any order or generation are of equal diameter and length, and are arranged in parallel, and the blood pressures at all of the junctions between specific orders of vessels are equal (49). In this simplified circuit, the flow rate in each element of order n is Q_{max}/N_n where Q_{max} is the total flow rate into the coronary arterial tree and N_n is the total number of vessels at order n. The Q_{max} is determined as the ratio of pressure drop and the equivalent resistance of the entire tree (49). The resistance, R, is computed using Poiseuille's equation ($R = \frac{128\mu l}{\pi D^4}$, where μ represents the viscosity of blood; and l and D represent the length and diameter of a vessel segment, respectively). The equivalent resistance of a crown or the entire tree is then determined by the summation of the vessel segments either in series or in a parallel arrangement.

Bootstrapped Method. The bootstrap is a powerful tool to check statistical inferences where the significance tests of regressions are questionable due to the fact that the error may not be normally distributed (50). This method is based on the resampling with the replacement of the original data to obtain the distribution of the desired statistics. Hence, in cases where the error might be heavy-tailed, it would provide to compare results with standard parametric assumptions. Specifically, we used this technique to test if the confidence intervals obtained for the asymmetric data sets are valid as the regressions visually seem better for the larger vessels. Since the morphometric data span several orders of magnitude, this method enables us to obtain the distribution of the scaling exponents, the standard error and the confidence interval of resampled data. We used 1,000 bootstrapped data sets resampled based on the bootstrap technique to calculate the confidence intervals, the standard error and the mean scaling exponents (please see Fig. S1). Further, the Pearson correlation coefficients for the flow-capillary, length-capillary and transit time-length and volume relations were presented, where linear relationships were hypothesized, to compare with the r-squared obtained from the least-square method for nonlinear regression (please see Fig. S2).

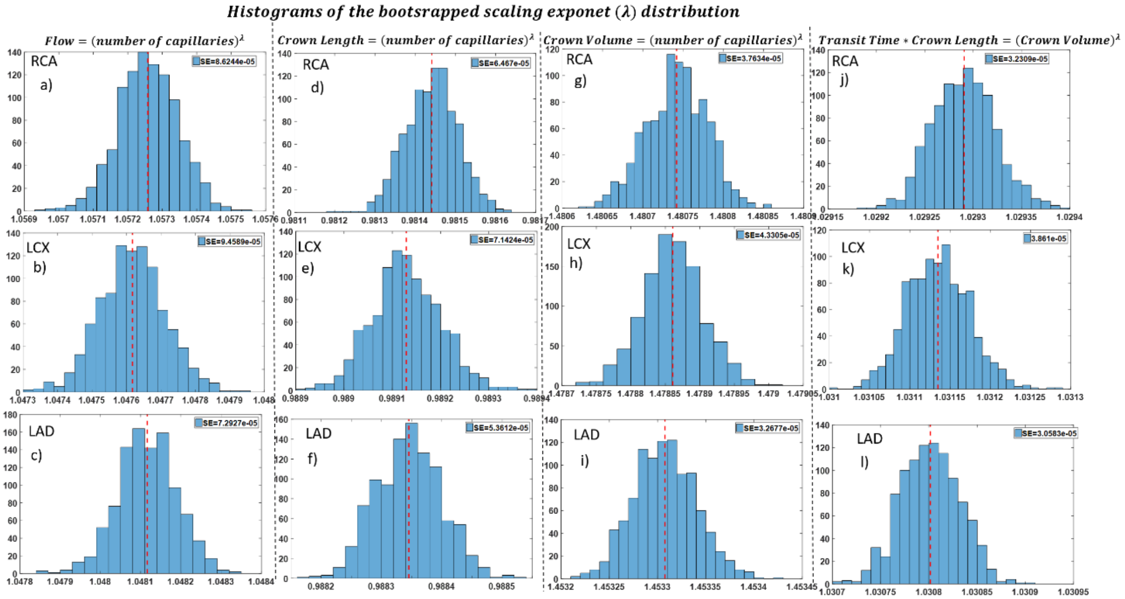

Figure S1. The histograms of the bootstrapped scaling exponents (λ) for the asymmetric data of pig. a,b,c) flow-capillary relation d,e,f) length-capillary relation g,h,i) volume-capillary relation j,k,l) transit time relationship with length and volume. The dashed lines show the mean scaling exponents, SE is the standard error shown in each panel, RCA, LCX, and LAD stand for right coronary artery, left circumflex artery, and left anterior descending artery respectively.

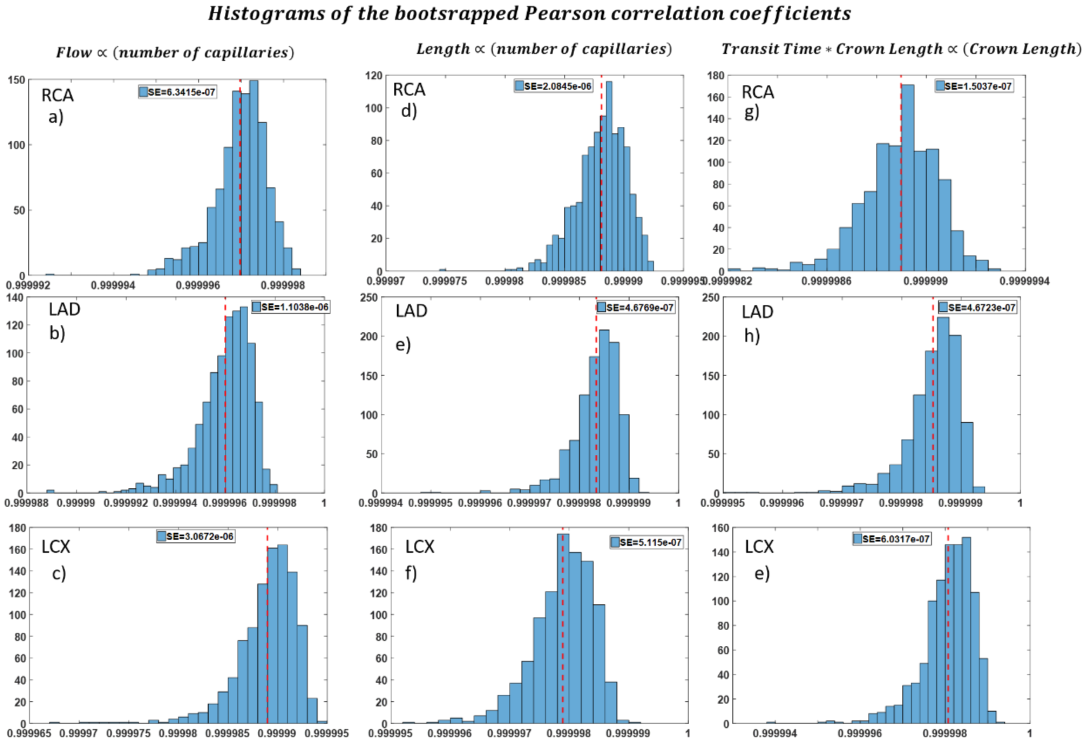

Figure S2. The histograms of the bootstrapped Pearson correlation coefficient for the asymmetric data of pig coronary arterial tree. a,b,c) linear flow-capillary relation d,e,f) linear length-capillary relation g,h,i) linear transit time relationship with length and volume. The dashed lines show the mean scaling exponents, SE is the standard error shown in each panel, RCA, LCX, and LAD stand for right coronary artery, left circumflex artery, and left anterior descending artery respectively.
